# Case of Remarkable Hypertrophy of the Fingers in a Girl; with a Notice of Some Similar Cases

**Published:** 1845-07

**Authors:** T. B. Curling

**Affiliations:** Lecturer on Surgery, and Assistant Surgeon to the London Hospital


*Case of remarkable hypertrophy of the fingers in a girl; with a notice of some similar cases.* By T. B. Curling, Lecturer on Surgery, and Assistant Surgeon to the London Hospital.—E. Hitchcock, aged 15, the subject of this congenital malformation is a pale sickly girl, the daughter of poor parents. On the right hand, the fore, middle and ring fingers are of unusual size. The enlargement of the fore and ring fingers is only slight, but the middle one is of extraordinary proportions, measuring as much as five inches and a half in length, and four in circumference. On the left hand, the thumb, index, and middle fingers, are hypertrophied. The index-finger, which is most enlarged, measures five inches and a quarter in length, and four in circumference. The middle finger has a lateral inclination, occasioned by the displaced extensor tendon, which forms a bridle along its outer edge. All parts of these hypertrophied fingers are equally developed in excess—the bones, articulations, integuments, and nails. The two largest fingers are fixed in an extended position, and the author attributes the girl's inability to bend them to the flexor mucles not having acquired a developement corresponding to the fingers upon which they act.

The author observes, that in this remarkable example of partial hypertrophy, there is an apparent absence of all those circumstances which seem favourable to excessive growth—a feeble constitution, sparing nutriment, no extraordinary exercise of the part, and no enlarged vessels, or activity of circulation. It would seem as if the formative powers which we see in some few cases exercised to excess in every part of the frame, so as to make a giant, had been limited in this instance, to an insignificant part of the extremities.

The paper contains a communication from Professor Owen, giving an account of a case analogous to the preceding one. The subject was a child, two years years old. The middle finger of each hand was nearly twice as long, and more than twice as thick, as the index-finger.

The author gives the particulars of a hand, a cast of which was given to him by Mr. Diamond of Frithstreet. It is from a Spaniard, the governor of a fort in the Philippine Islands, and presents congenital hypertrophy of the first and second fingers of the right hand. The second, which is of enormous size, had the same kind of lateral inclination as was observed in one of the fingers of Eliza Hitchcock. The author also describes a cast contained in the museum of King's College, London, of the hand of an adult in which the middle finger is hypertrophied, with a slight lateral inclination.

The author, after noticing the rarity of malformation, refers to two published cases of it—one recorded by Mr. Power of Dublin, the other by Dr. John Reid. In the former, the fingers were divaricated from the middle. In all the four foregoing cases in which the hypertrophied fingers were bent to one side, the author suspects that the inclination was produced, as in the case of Hitchcock, by the tension of the displaced extensor tendon, which had not elongated in proportion to the increase of the size of the finger.

The author notices whether it be possible, by any mode of treatment, to arrest the inordinate growth of the fingers in early life. He doubts if this could be accomplished by any other means than by firm and long-continued pressure; but, in addition to the suffering attending it, the impairment of the functions of the part caused by pressure constitutes an insuperable objection to its employment. In a case where one finger only was enlarged to a great extent, and nearly useless, he recommends its removal. In other cases, the enlarged finger might be reduced in size by amputation of the distal phalanx.

The paper is concluded by a brief notice of two cases of hypertrophy of the toes.—*Prov. Med. and Surg. Journal.*

